# A reduced order model formulation for left atrium flow: an atrial fibrillation case

**DOI:** 10.1007/s10237-024-01847-1

**Published:** 2024-05-16

**Authors:** Caterina Balzotti, Pierfrancesco Siena, Michele Girfoglio, Giovanni Stabile, Jorge Dueñas-Pamplona, José Sierra-Pallares, Ignacio Amat-Santos, Gianluigi Rozza

**Affiliations:** 1 Scuola Internazionale Superiore di Studi Avanzati (SISSA), Mathlab, Trieste, Italy; 2 The Biorobotics Institute, Sant’Anna School of Advanced Studies, Pisa, Italy; 3 Departamento de Ingeniería Energética, Universidad Politécnica de Madrid, Madrid, Spain; 4 Departamento de Ingeniería Energética y Fluidomecánica, Universidad de Valladolid, Valladolid, Spain; 5 Clinical University Hospital of Valladolid, Valladolid, Spain

**Keywords:** Reduced order model, Hemodynamics, Cardiovascular flows, Left atrium, Data-driven models, Patient-specific configurations

## Abstract

A data-driven reduced order model (ROM) based on a proper orthogonal decomposition-radial basis function (POD-RBF) approach is adopted in this paper for the analysis of blood flow dynamics in a patient-specific case of atrial fibrillation (AF). The full order model (FOM) is represented by incompressible Navier–Stokes equations, discretized with a finite volume (FV) approach. Both the Newtonian and the Casson’s constitutive laws are employed. The aim is to build a computational tool able to efficiently and accurately reconstruct the patterns of relevant hemodynamics indices related to the stasis of the blood in a physical parametrization framework including the cardiac output in the Newtonian case and also the plasma viscosity and the hematocrit in the non-Newtonian one. Many FOM-ROM comparisons are shown to analyze the performance of our approach as regards errors and computational speed-up.

## Introduction and motivation

Atrial fibrillation (AF) is the most common type of cardiac arrhythmia, affecting around 1.5% of the population (more than 35 million people worldwide Benjamin et al. (2019)). Its incidence seems to be correlated with age, since 8% of individuals above 80 years old are affected Go et al. (2001). An AF episode causes the Left Atrium (LA) to contract in an irregular and ineffective way, being typically triggered by irregular electrical impulses coming from the Pulmonary Vein (PV) roots. This abnormal contraction pattern seems to be related to stroke incidence due to thrombus formation within the Left Atrial Appendage (LAA) Wolf et al. (1991).

The LAA is a cavity which results from LA embryonic development, having a protruding and trabeculated morphology Al-Saady et al. (1999). When a patient develops AF, the LAA natural contractility reduces dramatically, making it prone to thrombi formation Seo et al. (2016); Goette et al. (2016). Due to this, the LAA has received a lot of attention from both clinical and biomedical engineering fields. Although a large number of studies have attempted to delve deeper into the relationship between stroke risk and LAA morphology in AF patients, the underlying mechanisms are still not well understood. Some of these previous studies have suggested certain morphologies to be associated with a lower risk of stroke Yaghi et al. (2020); Di Biase et al. (2012); Lee et al. (2015), while others could not find a correlation between the two phenomena Khurram et al. (2013); Nedios et al. (2014). Other clinical studies associated stroke risk with certain atrial geometric parameters such as LAA volume Korhonen et al. (2015); Beinart et al. (2011), LAA ostium area Lee et al. (2015, 2017); Beinart et al. (2011), number of lobes Yamamoto et al. (2014), and LAA depth Beinart et al. (2011). Recent studies also drew attention to the importance of the atrial flow Lee et al. (2017) and the PV morphology Polaczek et al. (2019). Although everything seems to point to the existence of a mechanistic relationship between the thrombosis risk and the LA anatomy and flow, this one remains unknown at this time.

Recent advances in medical imaging have made it possible to apply Computational Fluid Dynamics (CFD) techniques to the study of LA flows. Early research on LA flow dynamics was part of whole left-heart simulations Chnafa et al. (2014); Vedula et al. (2015). More recent CFD studies have presented numerical analyses of the LA flow patterns Chnafa et al. (2014); Otani et al. (2016); Lantz et al. (2019); Zingaro et al. (2021), focused on investigating blood stasis, as it is considered a necessary thrombogenic factor. Some of them also studied specifically the LAA stasis in AF conditions Masci et al. (2019); García-Isla et al. (2018); Bosi et al. (2018); García-Villalba et al. (2021); Dueñas-Pamplona et al. (2021, 2021); Musotto et al. (2022). All of them have helped to provide insights into the AF phenomenon and calculate otherwise inaccessible parameters such as residence times and shear stress that are related to blood stasis. Other studies have showed that the residence time and flow patterns for flexible-wall and rigid-wall simulations are very similar in case of impaired atrial function, especially when both the reservoir and booster functions are decreased García-Villalba et al. (2021); Dueñas-Pamplona et al. (2021). An interesting approach is used by Dueñas-Pamplona et al. (2022), who developed a morphing technique to study the risk of long-term stasis due to geometrical parameters. To reproduce the most critical AF case (in the absence of atrial contraction) the atrium was kept rigid, regardless of the atrial function at the time of medical imaging. Results show the enormous influence of cardiac output in the blood age indices, and the relatively minor role played by the PV orientation. To our knowledge, this is the only study that attempts to parametrize the LA flow problem. On the other hand, Gonzalo et al. (2022) have drawn attention to the fact that the non-Newtonian blood rheology can impact the left atrial stasis in patient-specific simulations, submitting that hematocrit-dependent non-Newtonian blood rheology should be considered when calculating patient-specific blood stasis indices by CFD Gonzalo et al. (2022).

Reduced order models (ROMs) Hesthaven et al. (2016); Benner et al. (2020a, 2020b, 2021); Rozza et al. (2023) have become increasingly important in hemodynamics applications due to the complex and multiscale nature of the cardiovascular system. ROM techniques allow for the creation of simplified models able to capture the essential features of the system and to significantly reduce the computational cost with respect to the standard CFD models based on classic discretization techniques, such finite volume (FV) or finite elements (FE) (hereinafter referred to as full order model (FOM)). ROMs can also aid in the development of personalized medicine, as they enable the creation of patient-specific models that can help clinicians to better understand and treat cardiovascular diseases.

Classic projection-based ROMs were already adopted to speed up patient-specific cases or idealized cardiovascular benchmarks. A proper orthogonal decomposition (POD)-Galerkin strategy is employed in combination with a FV full order solver in Buoso et al. (2019). The aim is to predict the pressure drop along an idealized vessel in a geometrical parameter setting. The same ROM approach but within a FE environment is adopted in Zainib et al. (2021); Ballarin et al. (2016, 2017) for the study of the blood flow patterns in patient-specific configurations of coronary artery bypass grafts where a physical parametrization involving the Reynolds number as well as an efficient centerlines-based geometrical parametrization are employed. For sake of completeness, concerning POD-based ROMs, we mention also earlier hemodynamics or electrophysiology papers which proposed preliminary geometry and/or physical parametrization studies Boulakia et al. (2012); Caiazzo et al. (2016); Guibert et al. (2014).

More recently, the combination of data-driven ROMs and FV method is becoming particularly appealing, due to the diffusion in the biomedical engineering community of commercial codes relying on FV schemes: see, e.g., Caruso et al. (2015); Vignali et al. (2021); Benim et al. (2011). In Girfoglio et al. (2022), a POD with interpolation by radial basis function (RBF) approach is adopted for the investigation of the hemodynamics in the aortic arch in the presence of a left ventricular assist device. The authors report a speed up of $\mathscr {O}(10^{6})$ associated with an error less than $15\%$ which represents a promising result in such a direction. For this reason, in Balzotti et al. (2022); Siena et al. (2023, 2023); Hesthaven and Ubbiali (2018) a similar approach is proposed, employing a feed-forward Neural Network for interpolation instead of relying on RBF in the analysis of the blood flow patterns in coronary artery bypass grafts. While in Balzotti et al. (2022) only one physical parameter is introduced (the Reynolds number), in Siena et al. (2023) a geometrical parametrization setting (with respect to the diameter of an isolated stenosis) is also considered. In such works, a speed up of $\mathscr {O}(10^{5})$ and an average error below $5\%$ are obtained.

Concerning the problem addressed in this paper, some recent works Saiz-Vivó et al. (2022); Pons et al. (2022) use machine learning-based models to infer LAA blood stasis from LAA geometry stasis. Their goal is to elaborate the huge amount of information coming from the data in order to identify patients with AF at the highest risk of thrombus formation. Unlike these works, mainly based on data processing, our aim is to retain the physical problem and build a cooperation between ROM, CFD and data-driven techniques in order to build an efficient computational tool able to achieve faithful solutions. Following this research line and its encouraging results, the present work tries to extend the use of data-driven ROM approaches to the study of the blood flow in the LAA portion in a physical parametrization setting. This represents a more intricate scenario due to the complexity of the geometry and of the flow, which is now fully three dimensional. So our work is an advancement compared to the vessel-like structures previously examined, where the blood flow follows mainly a unidirectional trajectory Balzotti et al. (2022); Siena et al. (2023, 2023); Girfoglio et al. (2021, 2022). Indeed, to the best of our knowledge, this work represents the first study about the application of ROM to the hemodynamics in the LA. For sake of completeness, we mention Fresca et al. (2021) where a ROM for the propagation of the electrical signal in the heart is analyzed. However, the authors do not address the fluid dynamics of the problem and furthermore use an idealized LA domain while a patient-specific one is used in this work, which represents an additional difficulty introduced in our research.

The paper is organized as follows. In Sect. 2 and 3, we describe in detail the adopted FOM and the ROM, respectively, along with the hemodynamics indices of interest and the techniques chosen for each algorithm. Then, Sect. 4 is reserved for error and efficiency analysis of our ROM approach. It also includes some FOM-ROM qualitative comparisons and clinical considerations on the patterns obtained. Finally, Sect. 5 is dedicated to draw conclusions and some possible extensions of this work.

## The full order model

The FOM employed in this paper is similar to the one proposed by Dueñas-Pamplona et al. (2021). It consists of parametrized Navier–Stokes equations for the blood flow in a patient-specific domain $\Omega$ over a time interval of interest $(t_0,T]$:

1 $$\begin{aligned} {\left\{ \begin{array}{ll} \rho \partial _t \textbf{v}(\textbf{x}, t)+ \rho \nabla \cdot (\textbf{v}(\textbf{x}, t) \otimes \textbf{v}(\textbf{x}, t)) - \nabla \cdot \mathbb T (\textbf{x}, t) = 0 &{} \quad \text {in} \quad \Omega \times (t_0,T],\\ \nabla \cdot \textbf{v}(\textbf{x}, t) = 0 &{} \quad \text {in} \quad \Omega \times (t_0, T], \end{array}\right. } \end{aligned}$$

where $\textbf{v} = \textbf{v}(\textbf{x}, t)$ and $p = p(\textbf{x}, t)$ are the velocity and the pressure. In addition, $\rho = 1050$ kg/m^3^ is the blood density. Problem (1) is endowed with initial data $\textbf{v}(\textbf{x}, t_0) = \textbf{0}$ and suitable boundary conditions reported in Sect. 2.1. $\mathbb T$ is the Cauchy stress tensor whose constitutive relation has the form:

2 $$\begin{aligned} \mathbb T =-p\mathbb I + \mathbb {T}_d, \end{aligned}$$

where the deviatoric component, $\mathbb {T}_d$, depends on the fluid model. It is known that, even if plasma is approximately Newtonian, whole blood could exhibit significant non-Newtonian features Chien et al. (1967). Many works compare Newtonian and non-Newtonian models, showing that the Newtonian one is in general admissible although non-Newtonian is considered to be more accurate Gonzalo et al. (2022). However, some differences can be found locally for some portions of the domain and/or during specific time instances of the cardiac cycle Johnston et al. (2004). For such a reason, in this work, both models, Newtonian and non-Newtonian, are considered.

For a Newtonian fluid, the tensor $\mathbb {T}_d$ is:

3 $$\begin{aligned} \mathbb {T}_d = 2\tilde{\mu }\mathbb {D(\textbf{v})}, \end{aligned}$$

with $\tilde{\mu } = 0.0035$ Pa$\cdot$s the constant dynamic viscosity and $\mathbb {D(\textbf{u})}=\frac{\nabla \textbf{u}+ \nabla \textbf{u}^T}{2}$ the strain rate tensor.

On the other hand, the non-Newtonian behavior of the blood is handled with the Casson’s model Drapaca et al. (2018):

4 $$\begin{aligned} \mathbb {T}_d =2\tilde{\mu }(J_2)\mathbb {D(\textbf{v})}, \end{aligned}$$

and

5 $$\begin{aligned} \tilde{\mu } (J_2) = \left[ (\bar{\eta }^2 J_2)^{1/4} + \Big (\frac{\tau _y}{2}\Big )^{1/2} \right] ^{1/2} J_2^{-1/2}, \end{aligned}$$

where $J_2$ is the second invariant of $\mathbb {D(\textbf{v})}$, $\bar{\eta }= \eta / (1- H )^{2.5}$ with $\eta$ the plasma viscosity and *H* the hematocrit, and $\tau _y = (a_1 +a_2 H )^3$ where for blood $a_1=0$ and $a_2=0.290$ Pa^1/3^Fung (1993). For more details we refer to Errill (1969). We note that the plasma viscosity $\eta$ and the hematocrit *H* will be treated as parameters in our ROM framework.

Equations above are solved using the FV method implemented in OpenFOAM(R) 2206 OpenFOAM Library (2022). Direct numerical simulation of the blood flow is used here assuming that the flow is not turbulent. This assumption is based on the work of Dueñas-Pamplona et al. (2022) in which negligible differences between mean flow fields of turbulent and non-turbulent simulations were found. A second-order Gauss linearUpwind scheme Warming and Beam (1976) is adopted for space discretization, and first-order Euler implicit time scheme is used for time discretization. More details about the FV discretization of Navier–Stokes equations can be found in Girfoglio et al. (2022).

### Patient-specific geometry and boundary conditions

**Fig. 1:**
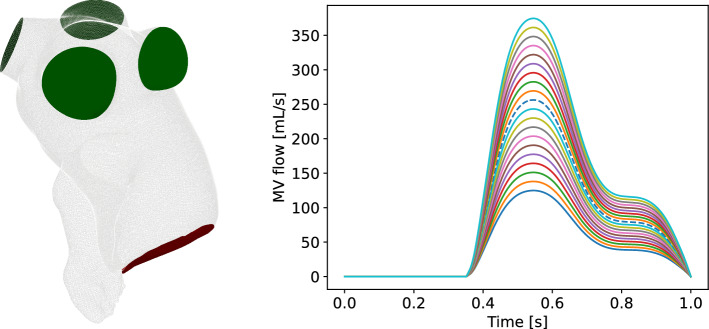
Left: LA patient-specific geometry; the green surfaces are the PV inlets, while the red region is the MV outlet. Right: boundary conditions for MV flow for different scaling factors ranging in [0.5, 1.5] (the dashed line corresponds to 1)

In this research, the same patient geometry reported by (Dueñas-Pamplona et al. 2022) is used as test configuration. The patient had a history of paroxysmal AF, but no previous stroke or transient ischemic attack, and underwent CT-imaging and Doppler transesophageal echocardiography at the Puerta de Hierro Hospital in Madrid. The images were obtained from the left atrial endocardial surface, including the LAA and the PVs, during normal sinus rhythm, using a SIEMENS Sensation 64 scanner with specific scanning parameters. The resulting DICOM images were manually segmented with the aid of the non-commercial code 3D-Slicer to extract the three-dimensional surface of the endocardium. The PVs were removed beyond the first bifurcation using Autodesk MeshMixer, and the LA endocardial surface geometry, including the LAA, was provided. In Fig. 1 (left), the patient-specific geometry is reported.

Walls are assumed to be completely rigid due to the patient’s condition. This choice is justified by recent studies García-Isla et al. (2018); Dueñas-Pamplona et al. (2021) that have compared the results of the fixed wall and moving wall simulations, showing that both approaches develop flow patterns and residence time distributions very similar when dealing in cases of impaired function, especially when both the reservoir and booster functions of the appendage are decreased. Regarding inflow and outflow boundary conditions, those reported in Dueñas-Pamplona et al. (2022) are also used. They consist of Mitral Valve (MV) velocities during sinus rhythm after CT imaging such velocities were obtained by echo Doppler, scaling it by a factor to check the effects of varying cardiac output. These blood velocities were used to establish the simulation boundary conditions, assuming each PV carries out the same flow-rate. In order to clarify the inflow and outflow boundaries, in Fig. 1 (left) are shown in red and green, respectively, the MV and the PVs. In addition, in the right panel, we report the time evolution of the MV flow-rate for different values of the scaling factor *f* in the interval [0.5, 1.5]. Note that the scaling factor *f* belongs to the parameter space of our problem.

### Hemodynamics indices

Now we are going to introduce some relevant indices associated with the stasis of the blood flow Dueñas-Pamplona et al. (2021). They represent fundamental quantities for the medical community because high residence times of the blood flow are related to thrombus formation.

#### Age distribution

Following Sierra-Pallares et al. (2017), the first and second moments of age, $m_1$ and $m_2$, are computed as follows:

6 $$\begin{aligned} m_k(\textbf{x}, t)= \int _{-\infty }^{t} t^k\phi (\textbf{x}, t) dt, \quad k=1,2, \end{aligned}$$

where $\phi (\textbf{x}, t)$ is the blood age distribution. In particular, $m_1$ represents the mean age of blood and describes the time needed for a given blood particle to reach another position in the computational domain, so large values of $m_1$ denote stasis of the blood. The second moment $m_2$ does not have any physical meaning. However, we have decided to consider it as a further variable to be taken into account to show the versatility of our ROM approach. It should be noted that once $m_k$ is known, it would be possible to compute also $\phi$ (6): see, e.g., John et al. (2007); Sierra-Pallares et al. (2017). For a laminar flow, we have for $k = 1, 2$:

7 $$\begin{aligned} {\left\{ \begin{array}{ll} \rho \frac{\partial m_k}{\partial t} + \rho \nabla \cdot (\textbf{v} m_k) = \nabla \cdot \big [ \mu _{m_k} \nabla m_k\big ] + \rho k m_{k-1} &{} \quad \text {in} \quad \Omega \times (t_0, T], \\ m_k = 0 &{} \quad \text {on} \quad \Gamma _i \times (t_0, T], \\ \nabla m_k \cdot \textbf{n} = 0, &{}\quad \text {on} \quad \partial \Omega \smallsetminus \Gamma _i \times (t_0, T], \end{array}\right. } \end{aligned}$$

and initial data $m_k = 0$, where $\textbf{n}$ is the unit normal outward vector to the boundary, $\Gamma _i$ denotes the inflow boundaries (i.e., the PV inlets, see Fig. 1), $\mu _{m_k} = 10^{-10}\, \text {kg}/(\text {m} \cdot \text {s})$ is the mass diffusivity of the moment $m_k$ Dueñas-Pamplona et al. (2021) and $m_0=1$ Sierra-Pallares et al. (2017).

Another variable related to the blood age is the washout. It is computed by solving a scalar transport equation similar to (7) but without the source term

8 $$\begin{aligned} {\left\{ \begin{array}{ll} \rho \frac{\partial \varphi }{\partial t} + \rho \nabla \cdot (\textbf{v} \varphi ) = \nabla \cdot \big [ \mu _{\varphi } \nabla \varphi \big ] &{} \quad \text {in} \quad \Omega \times (t_0, T], \\ \varphi = 0 &{} \quad \text {on} \quad \Gamma _i \times (t_0, T], \\ \nabla \varphi \cdot \textbf{n} = 0 &{}\quad \text {on} \quad \partial \Omega \smallsetminus \Gamma _i \times (t_0, T], \end{array}\right. } \end{aligned}$$

and initial data $\varphi = 1$, where $\mu _{\varphi } = 10^{-10} \text {kg}/(\text {m} \cdot \text {s})$. The washout is the residual value of $\varphi$ as the dynamics evolves over the cardiac cycle. It implies zones with high stasis and therefore high age. More details could be found in John et al. (2007); Sierra-Pallares et al. (2017).

Equations (7) and (8) are coupled with system (1) but, in order to reduce the computational cost, we adopt a segregated algorithm, so they are solved *after* the system (1). In other words, $m_k$ and $\varphi$ are treated as passive scalars. Equations (7) and (8) are employed by using second-order schemes for space discretization and Euler scheme for time discretization.

#### Wall shear stress and oscillating shear index

The Wall Shear Stress (WSS) can be defined as follows:

9 $$\begin{aligned} \text {WSS} = \mathbb {T}_d \cdot \textbf{n}, \quad \text{ on } \quad \partial \Omega . \end{aligned}$$

The interest is on the Time-Averaged Wall Shear Stress (TAWSS) representing the mean effect of the WSS on the entire cardiac cycle:

10 $$\begin{aligned} \text {TAWSS} = \frac{1}{T}\int _0^T \Vert \text {WSS}\Vert dt. \end{aligned}$$

Low values of TAWSS correspond to stasis regions.

Another important quantity is the Oscillating Shear Index (OSI):

11 $$\begin{aligned} \text {OSI} = \frac{1}{2} \left[ 1 - \frac{\Vert \int _0^T \text {WSS}dt\Vert }{\int _0^T \Vert \text {WSS}\Vert dt} \right] . \end{aligned}$$

It is related to the oscillations of the flow and ranges from 0, when the flow is unidirectional, to 0.5, when the direction of the flow is totally reversed. The OSI is a useful indicator in cardiovascular problems because it is correlated with the intimal thickness of the wall and the restenosis process Ku et al. (1985).

## The reduced order model

The basic assumption of ROM for a partial differential equations problem depending on time *t* and parameter vector $\varvec{\mu }$ is that any solution can be represented as a linear combination of a reduced number of global basis functions, that depend exclusively on the space $\textbf{x}$, with the weights of the linear combination depending only on *t* and $\varvec{\mu }$. For a generic variable $\Phi$, this is written as:

12 $$\begin{aligned} \Phi (\textbf{x}, t; \varvec{\mu }) \approx \Phi ^{\textrm{rb}}(\textbf{x}, t; \varvec{\mu }) = \sum _{l=1}^{L}\alpha _{l}(t, \varvec{\mu })\varvec{\ell }_{l} (\textbf{x}), \end{aligned}$$

where $\Phi ^{\text {rb}}$ is the reduced order approximation of $\Phi$, *L* is the number of basis functions, the $\varvec{\ell }_l$ are the basis functions and the $\alpha _l$ are the weights of the linear combination (the so-called modal coefficients). In this work, we are interested in the reconstruction of all the hemodynamics indices introduced in Sect. 2.2, so we have $\Phi = \{m_1, m_2, \varphi , \text {TAWSS}, \text {OSI}\}$. It should be noted that TAWSS and OSI are steady-state variables. In this case, Eq. (12) becomes:

13 $$\begin{aligned} \Phi (\textbf{x}; \varvec{\mu }) \approx \Phi ^{\textrm{rb}}(\textbf{x}; \varvec{\mu }) = \sum _{l=1}^{L}\alpha _{l}(\varvec{\mu })\varvec{\ell }_{l} (\textbf{x}). \end{aligned}$$

However, for the sake of good order, in the following explanation, we refer to a time and parameter-dependent variable.

We use the POD-RBF technique, which is divided into the following two phases:

Offline: given a set of physical parameter values and time instances, the corresponding high-fidelity solutions (the so-called snapshots) are computed and collected into a matrix. The POD algorithm is used to extract the reduced basis space from the snapshots matrix. Then, the snapshots are projected onto the POD space by obtaining the corresponding modal coefficients. Finally, a RBF interpolation algorithm is used to compute a map between the parameters and the modal coefficients.
Online: given a set of new physical parameter values, the corresponding modal coefficients are computed via the RBF function and the approximated solution is recovered as a linear combination between these coefficients and the POD reduced basis (see Eq. (12)).

The offline–online procedure is summarized in Algorithm 1. The implementation of the ROM relies on the Python library EZyRB Demo et al. (2018).

**Algorithm 1:**
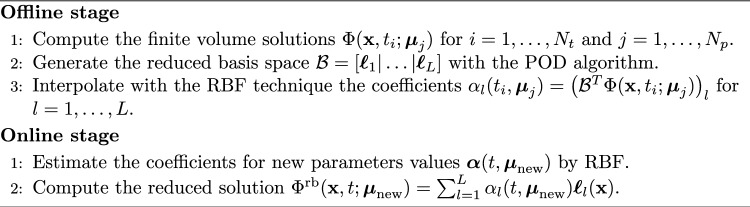
Description of the main steps of the Offline/Online framework

### The proper orthogonal decomposition

Let $\mathcal {K} = \{\varvec{\mu }_1, \dots , \varvec{\mu }_{N_p}\}$ be a finite dimensional training set of samples chosen inside the parameter space $\mathcal P$, and let $\mathcal {T} = \{t_1, \dots , t_{N_t}\} \subseteq (t_0, T]$ be the time discretization. We refer to the FOM snapshots $\Phi (\textbf{x}, t_i; \varvec{\mu }_j)$ for $i = 1,\dots , N_t$ and $j = 1,\dots , N_p$.

The POD Atwell and King (2001); Gunzburger (2002); Kunisch and Volkwein (2002); Volkwein (2011); Willcox and Peraire (2002) is one of the most common techniques used to extract the essential information from the space generated by the solution manifold. Let $N_{s} = N_t \cdot N_p$ be the dimension of this space: The goal of the POD is to construct a reduced basis space of dimension $L\ll N_{s}$ which is optimal in the least-square sense. More precisely, the POD algorithm builds the reduced basis space which minimizes the quantity

$$\begin{aligned} \sqrt{\sum _{(t, \varvec{\mu })\in \mathcal {T} \times \mathcal {K}}\inf _{\Phi ^{\textrm{rb}}\in V^{\textrm{rb}}}\Vert \Phi (\textbf{x}, t; \varvec{\mu })-\Phi ^{\textrm{rb}}(\textbf{x}, t; \varvec{\mu })\Vert ^{2}}, \end{aligned}$$

among all *L*-dimensional subspaces $V^{\textrm{rb}}$ spanned by the FOM solutions Hesthaven et al. (2016).

Let $N_{h}$ be the number of the mesh cells. We collect the FOM solutions into the snapshot matrix $S^{\Phi }$ given by

14 $$\begin{aligned} S^{\Phi } = \begin{pmatrix} \Phi _{1}(\textbf{x}, t_{1};\varvec{\mu }_{1}) &{} \dots &{} \Phi _{1}(\textbf{x},t_{N_t};\varvec{\mu }_{1}) &{} \Phi _{1}(\textbf{x}, t_{1};\varvec{\mu }_{2}) &{} \dots &{} \Phi _{1}(\textbf{x},t_{N_t};\varvec{\mu }_{N_p})\\ \Phi _{2}(\textbf{x},t_{1};\varvec{\mu }_{1}) &{} \dots &{} \Phi _{2}(\textbf{x},t_{N_t};\varvec{\mu }_{1}) &{} \Phi _{2}(\textbf{x},t_{1};\varvec{\mu }_{2}) &{} \dots &{} \Phi _{2}(\textbf{x}, t_{N_t}; \varvec{\mu }_{N_p})\\ \vdots &{} \ddots &{} \vdots &{} \vdots &{} \ddots &{} \vdots \\ \Phi _{N_h}(\textbf{x}, t_{1};\varvec{\mu }_{1}) &{} \dots &{} \Phi _{N_h}(\textbf{x}, t_{N_t};\varvec{\mu }_{1}) &{} \Phi _{N_h}(\textbf{x}, t_{1};\varvec{\mu }_{2}) &{} \dots &{} \Phi _{N_h}(\textbf{x}, t_{N_t};\varvec{\mu }_{N_p})\\ \end{pmatrix}, \end{aligned}$$

whose dimension is $N_{h}\times N_{s}$. Since $S^{\Phi }$ is usually not squared, we introduce its rank $R\le \min \{ N_{h}, N_{s}\}$. By applying the Singular Value Decomposition (SVD) to $S^{\Phi }$, we can rewrite it as

$$\begin{aligned} S^{\Phi } = \mathcal {L} \Sigma \mathcal {R}^{T}, \end{aligned}$$

where $\mathcal {L} = [\varvec{\ell }_{1}| \dots |\varvec{\ell }_{N_{h}}] \in \mathbb {R}^{N_{h}\times N_{h}}$ and $\mathcal {R}= [\varvec{r}_{1}| \dots |\varvec{r}_{N_{s}} ] \in \mathbb {R}^{N_{s}\times N_{s}}$ are orthogonal matrices whose columns are the left and right singular vectors, respectively, and $\Sigma \in \mathbb {R}^{N_{h}\times N_{s}}$ is a diagonal matrix with *R* nonzero real singular values $\sigma _{1}\ge \sigma _{2} \ge \dots \ge \sigma _{R} > 0$.

As the rank *R* is typically large, we are now concerned with reducing the size of the problem. We rely on the Schmidt–Eckart–Young theorem Eckart and Young (1936), which states that the first *L* left singular vectors of $S^{\Phi }$ are the POD bases of rank *L*, with $L<R$. Hence, the POD bases are the first *L* columns of the matrix $\mathcal {L}$. In particular, the *l*-th column of $\mathcal {L}$ is the eigenvector associated with $\mathcal {C}\varvec{\ell }_{l}=\sigma _{l}^{2}\varvec{\ell }_{l}$, where $\mathcal {C}=(S^{\Phi })^{T}S^{\Phi }$ is the snapshot correlation matrix. Therefore, $\varvec{\ell }_{l}$ is given by

$$\begin{aligned} \varvec{\ell }_{l} = \frac{1}{\sigma _{l}}S^{\Phi }\varvec{c}_{l}, \quad \text {for} \quad l=1,\dots , L. \end{aligned}$$

Then, the POD bases, also known as modes, are collected into the matrix

15 $$\begin{aligned} \mathcal {B} = [\varvec{\ell }_{1}|\dots |\varvec{\ell }_{L}]. \end{aligned}$$

It remains only to choose properly the value of *L*. A common choice is to define it as the smallest integer such that

16 $$\begin{aligned} \frac{\sum _{l=1}^{L}\sigma _{l}^{2}}{\sum _{l=1}^{R}\sigma _{l}^{2}}\ge \varepsilon , \end{aligned}$$

for a given threshold $\varepsilon$ on the cumulative energy of the eigenvectors.

Once defined the POD basis matrix $\mathcal {B}$, the reduced solution $\Phi ^{\textrm{rb}}$ that approximates the truth one $\Phi$ is given by

$$\begin{aligned} \Phi ^{\textrm{rb}}(\textbf{x}, t_i; \varvec{\mu }_j) = \sum _{l=1}^{L}\alpha _{l}(t_i, \varvec{\mu }_j)\varvec{\ell }_{l}(\textbf{x}), \quad \text {for} \quad i = 1,\dots , N_t \quad \text {and} \quad j = 1,\dots , N_p \end{aligned}$$

and where $\alpha _{l}(t_i, \varvec{\mu }_j) = \left( \mathcal {B}^{T}\Phi (t_i, \varvec{\mu }_j)\right) _{l}$ is the *l*-th modal coefficient.

### Radial basis function interpolation

The last step of the offline phase consists of the definition of a map $F:\mathcal {T} \times \mathcal {K} \mapsto \mathbb {R}^{L}$ from the parameters space to the modal coefficients one. To this end, we use the RBF interpolation Buhmann (2003); Forti and Rozza (2014); Škala (2016). In the data-driven ROM framework, the approximation using RBF is an extremely powerful method due to its capability to produce smooth output maps from a large number of data points. Moreover, it has a simple architecture and has few parameters to be tuned compared to neural networks.

In general given a set of $N_s$ couples $(\varvec{x}_i, \varvec{y}_i)$ and a query point $\varvec{x}$, the RBF is defined as

17 $$\begin{aligned} F(\varvec{x})&= \sum _{j=1}^{N_{s}}w_{j}\widetilde{\psi }(\Vert \varvec{x}-\varvec{x}_{j}\Vert )\nonumber \\&+P(\varvec{x}), \quad \text {subject to }F(\varvec{x}_{i})=\varvec{y}_{i} \text { for } i=1,\dots ,N_{s}, \end{aligned}$$

where $w_{j}$ are weights to be determined and $P(\varvec{x})$ is a polynomial required for stability reasons. For simplicity, we assume that *P* is of degree 1. By adding the conditions

$$\begin{aligned} \sum _{j=1}^{N_{s}}w_{j} = 0\qquad \text {and}\qquad \sum _{j=1}^{N_{s}}w_{j}\varvec{x}_{j} = 0, \end{aligned}$$

the weights $w_{j}$ and the polynomial *P* are uniquely determined.

As explained in Sect. 3.1, the projection of the snapshots onto the POD space gives us the modal coefficients $\varvec{\alpha }(t_i, \varvec{\mu }_j) = \{\alpha _{l}(t_i, \varvec{\mu }_j)\}_{l=1}^L$ corresponding to the samples $(t_i, \varvec{\mu }_j)$, with $i=1,\dots ,N_t$ and $j=1,\dots ,N_p$. Thus, in our case, we have that $(\varvec{x}, \varvec{y}) \equiv \left( (t, \varvec{\mu }), \varvec{\alpha }(t, \varvec{\mu })\right)$.

## Numerical results

In this section, we investigate the performance of our ROM. For what concerns the validation of the FOM in an artificial setup and the independence of the numerical results from the mesh for the patient-specific case of interest, we refer to Appendix 6.

**Fig. 2:**
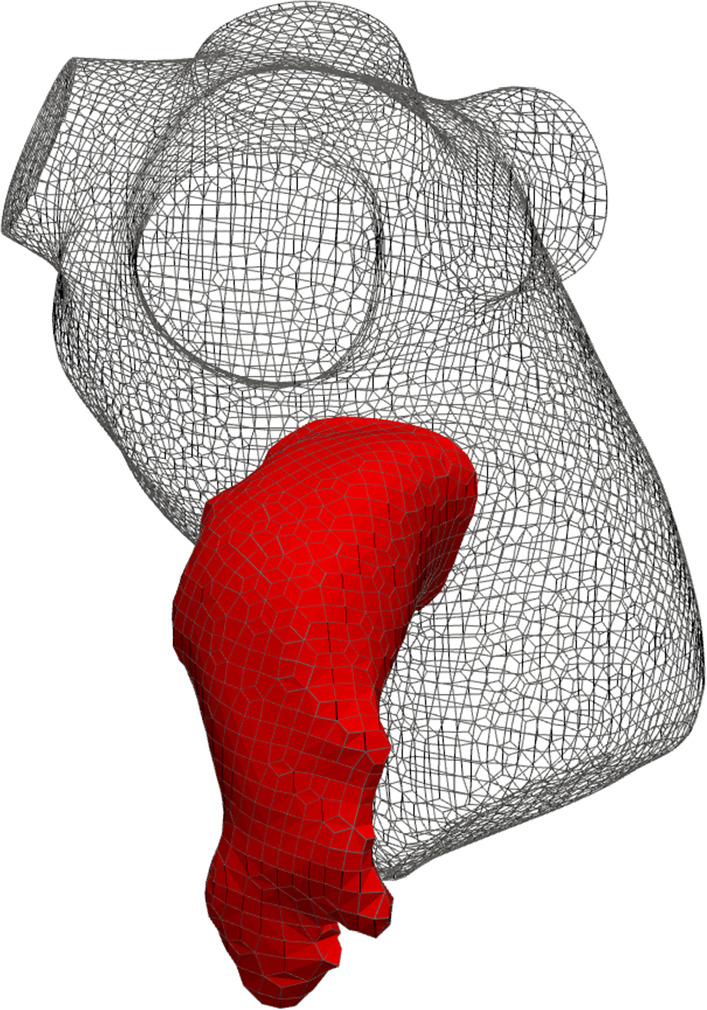
Sketch of the mesh. The red portion corresponds to LAA

We consider a tetrahedral mesh inside the domain $\Omega$ and on its boundary $\partial \Omega$, consisting of 542.560 cells and 79.708 cells, respectively, i.e., the Grid 2 reported in Table 2. In Fig. 2, we show a sketch of the mesh and we highlight the LAA portion on which we will focus our tests. (The reason for this choice is discussed in Sect. 1.) Since we are going to analyze the regime behavior, i.e., downstream of the transient effects, we collect the FOM solutions corresponding to the fourth cardiac cycle whose period is 1.07 s. Therefore, the effective time interval is $(t_{0},T] = (4.33, 5.4]$ s which, for sake of convenience, we report to (0, 1]. We ran the FOM simulations with a maximum time step $\Delta t=0.01\,$s and a maximum Courant number of 0.8, collecting $N_t = 107$ time-dependent snapshots.

To unify the notation, where necessary, hereinafter we will use the superscript *N* to refer to the Newtonian model and the superscript *C* to refer to the Casson’s one. In the Newtonian case we only have one parameter represented by the scaling factor *f* of the cardiac output, ranging in the interval [0.5, 1.5] (see Fig. 1), i.e., $\varvec{\mu }^N = f$. On the other hand, in the non-Newtonian case, we also consider the plasma viscosity $\eta \in [1.5\text {e}-03, 1.7\text {e}-03]$ and the hematocrit $H \in [35, 50]$, i.e., $\varvec{\mu }^C = \{f, \eta , H \}$. Specifically, the discrete set of $\varvec{\mu }^N$ consists of 20 points obtained by a uniform sampling procedure. Instead, for the Casson’s model, we get a distribution of 30 points for $\varvec{\mu }^C$ using Latin hypercube sampling McKay et al. (2000). Thus, we collect $20N_t = 2140$ snapshots for the Newtonian model and $30N_t = 3210$ snapshots for the Casson’s model.

For both models, we split the initial database into a training set $\mathcal {K}_{\textrm{train}}\subset \mathcal {K}$ and in a validation set $\mathcal {K}_{\textrm{test}} = \mathcal {K}\setminus \mathcal {K}_\textrm{train}$. All the snapshots belonging to the training set are stored in the matrix $S^\Phi$. The validation set is used to assess the accuracy of the ROM solution. In order to compare the results obtained with the two models, we choose similar test values for the scaling factor *f*, which is the only shared parameter. Therefore, we set $\mathcal {K}_\textrm{test}^N=\{\varvec{\mu }^N_3,\varvec{\mu }^N_{7}\}$ and $\mathcal {K}_\textrm{test}^C=\{\varvec{\mu }^C_{1},\varvec{\mu }^C_{14}\}$, where $\varvec{\mu }^N_3=0.65$, $\varvec{\mu }^M_7=0.87$, $\varvec{\mu }^C_1=[0.65, 39.7, 1.62\textrm{e}-03]$ and $\varvec{\mu }^C_{14}=[0.88, 46.7, 1.66\textrm{e}-03]$. Consequently, the Newtonian model is trained with $18 N_t = 1926$ snapshots while the Casson’s model with $28 N_t = 2996$ snapshots. It should be noted that we have tried to increase furthermore the size of the training set but no significant change in the computation of the POD basis has been appreciated.

To analyze the accuracy of the ROM we compute the relative error

18 $$\begin{aligned} \textsf{e}(t, \varvec{\mu }) = \frac{\Vert \Phi (t,\varvec{\mu })-\Phi ^{\textrm{rb}}(t,\varvec{\mu })\Vert }{\Vert \Phi (t,\varvec{\mu })\Vert }, \end{aligned}$$

where $\varvec{\mu }\in \mathcal {K}_{\textrm{test}}$ and $\Vert \cdot \Vert$ is the Frobenius norm. For the steady-state variables, $\text {TAWSS}$ and $\text {OSI}$, the relative error (18) becomes:

19 $$\begin{aligned} \textsf{e}(\varvec{\mu }) = \frac{\Vert \Phi (\varvec{\mu })-\Phi ^{\textrm{rb}}(\varvec{\mu })\Vert }{\Vert \Phi (\varvec{\mu })\Vert }. \end{aligned}$$

### Choice of the number of modes

**Fig. 3:**
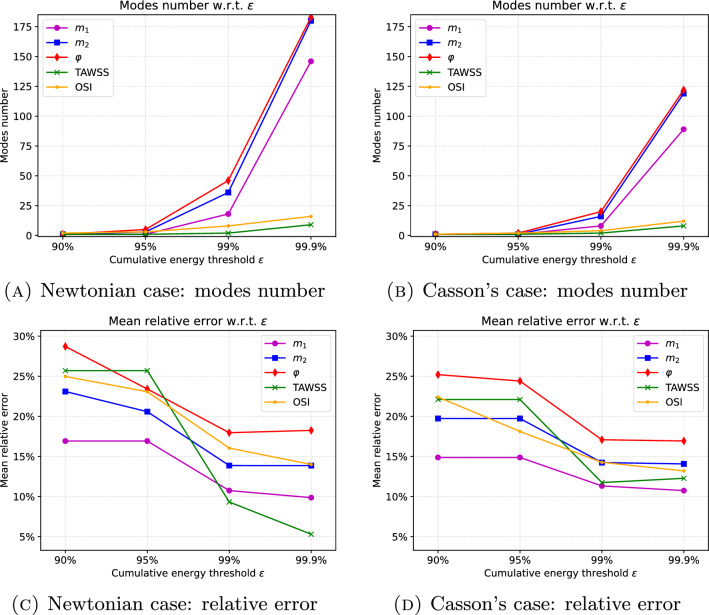
Variation of the number of modes and of the relative error with respect to the cumulative energy threshold $\varepsilon$ for the Newtonian (first column) and non-Newtonian (second column) case for all the variables involved

As explained in Sect. 3.1, the number of modes *L* is generally selected through the cumulative energy threshold $\varepsilon$ in Eq. (16) affecting the accuracy of the ROM approximation. In Fig. 3, we plot the modes number as well as the relative error $\textsf{e}$ (see Eqs. (18) and (19)), which is time average for $m_1$, $m_2$ and $\varphi$, against the value of $\varepsilon$ = $\{90\%, 95\%, 99\%,99.9\%\}$, for both the Newtonian and Casson’s models. As expected, the relative error decreases at increasing of modes number, i.e., for higher values of $\varepsilon$. However for $\varepsilon \ge 99\%$ the relative error reaches a plateau for most of the variables. On the contrary, the number of modes increases. Such increase is particularly pronounced for $m_1$, $m_2$ and $\varphi$ (and appears more evident in the Newtonian case, although the error level exhibited by the two models is comparable).

This could be due to the fact that TAWSS and OSI derive from an average in time over the cardiac cycle while the other indices are time-dependent and therefore characterized by a richer modal content. We fix the value of $\varepsilon$ to $99\%$ for the following tests, as it guarantees a good accuracy and at the same time it allows to monitor the computational cost.

### ROM solutions

**Fig. 4:**
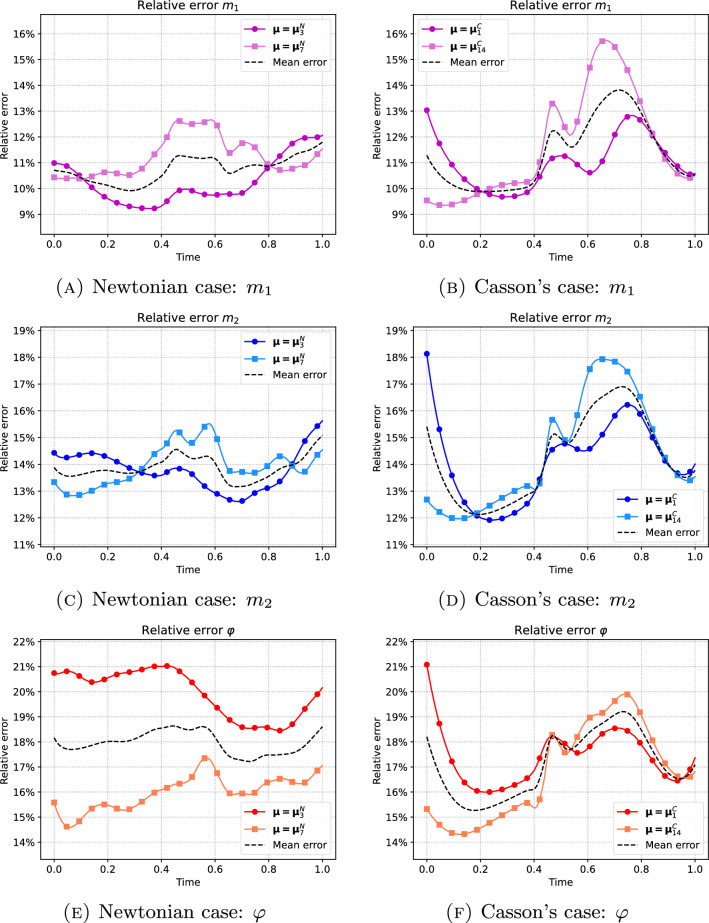
Variation in time of the relative error corresponding to the test parameters values for the Newtonian (first column) and non-Newtonian (second column) case for $m_1$, $m_2$ and $\varphi$

Once set the cumulative energy threshold $\varepsilon =99\%$, we proceed with the ROM simulations. In Fig. 4, we show the variation in time of the relative error $\textsf{e}(t,\varvec{\mu })$ defined in Eq. (18) for $m_1$, $m_2$ and $\varphi$ associated with the parameters values in $\mathcal {K}_\textrm{test}^N$ and $\mathcal {K}_\textrm{test}^C$, together with their mean values. Overall, we observe that the relative errors vary between 9% and 21% demonstrating a clinical relevance. More specifically, in the Newtonian case (left plots), the relative errors are quite similar to each other. They, along with the associated mean value, do not exhibit large oscillations during the cardiac cycle. For the Casson’s model (right plots) we observe that the error increases immediately after the opening of the MV (happening around $t=0.4$, see Fig. 1), so unlike the Newtonian case the error is affected by wide oscillations. We also note the increase of the error during the first few time steps which might be due to the transient nature of the flow, as also noted in other configurations including academic benchmarks Girfoglio et al. (2019).

**Fig. 5:**
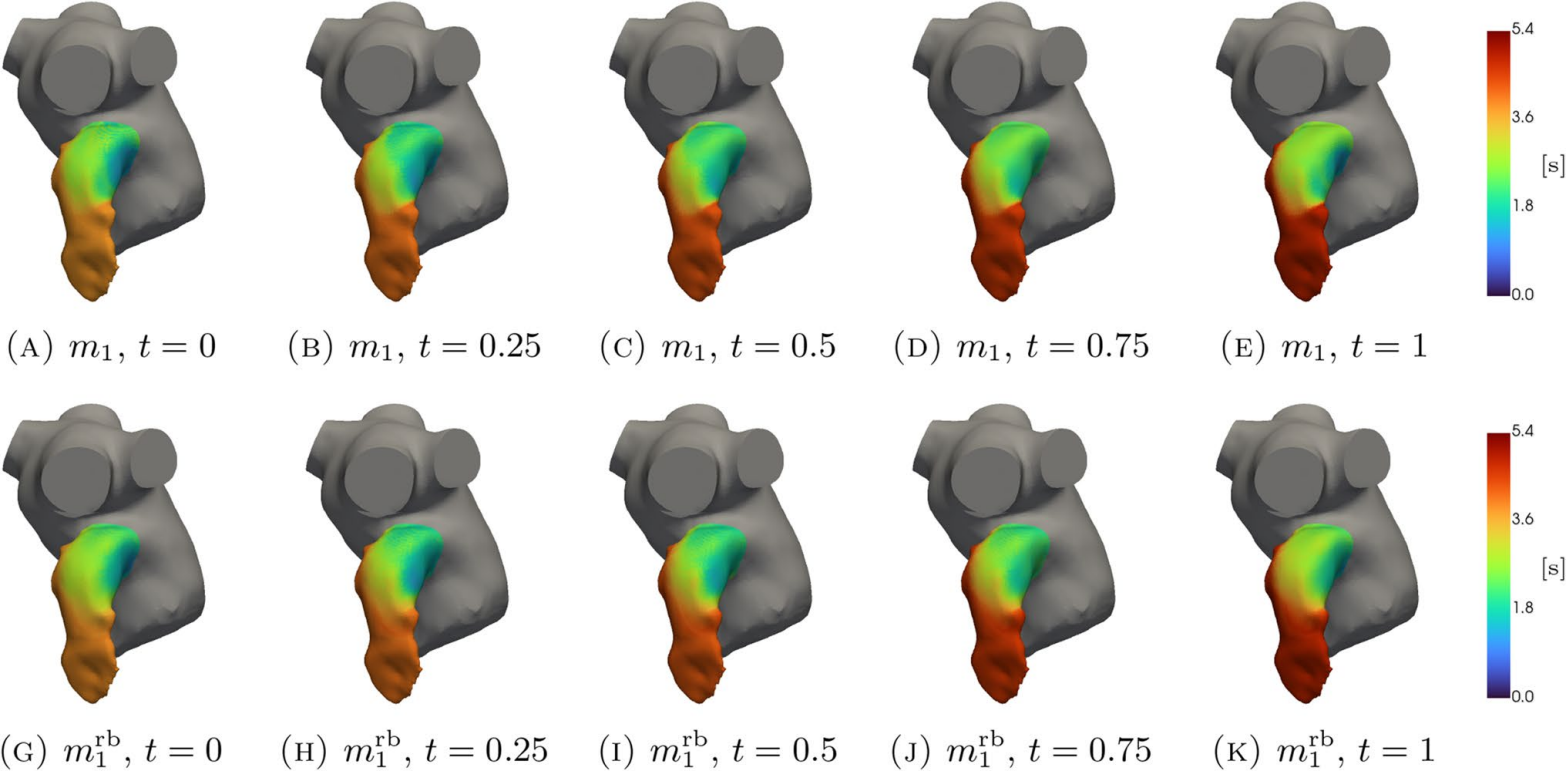
Newtonian case: qualitative comparison between FOM (top) and ROM (bottom) solutions at different times for $m_1$

**Fig. 6:**
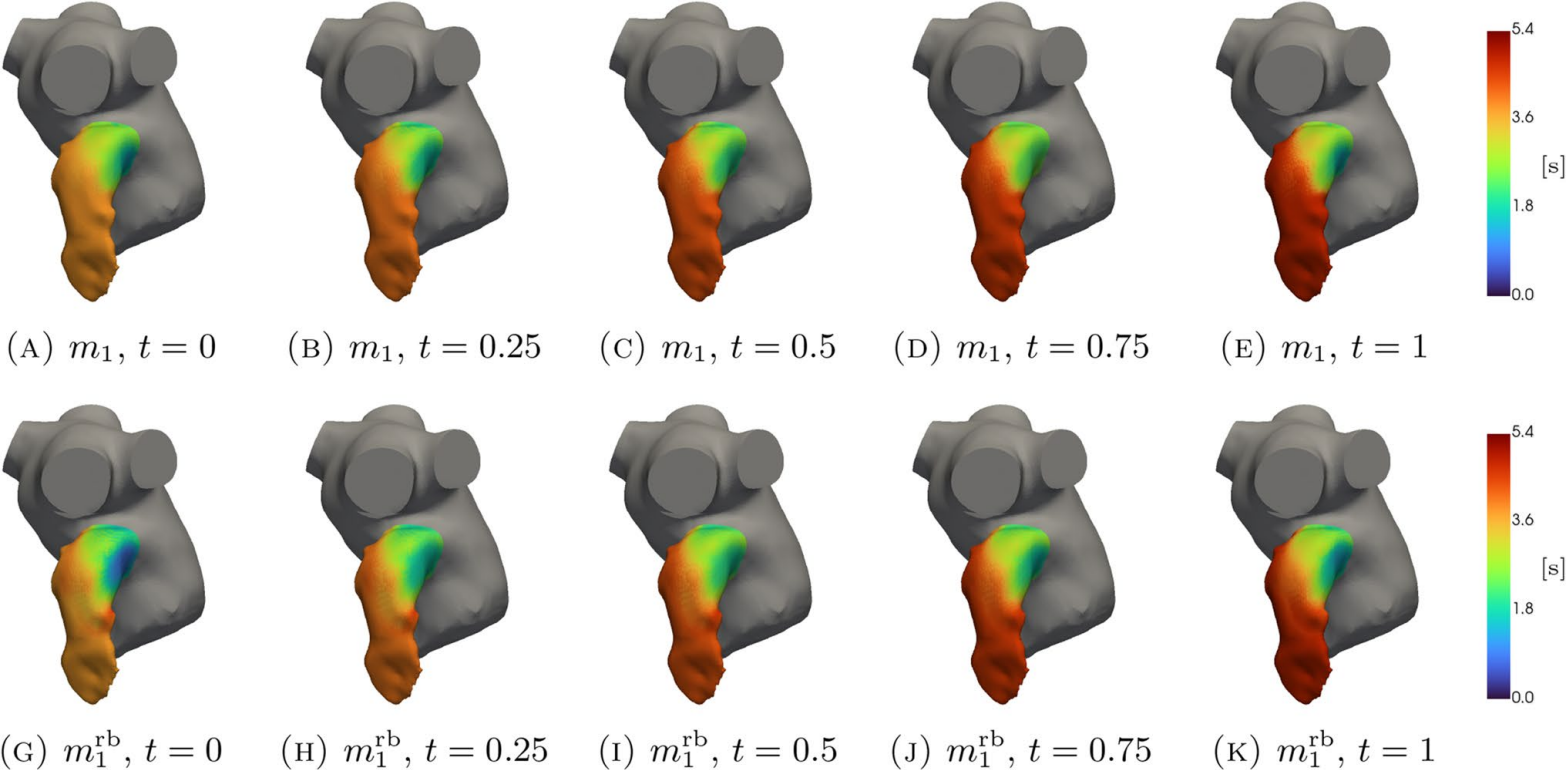
Casson’s case: qualitative comparison between FOM (top) and ROM (bottom) solutions, different times for $m_1$

**Fig. 7:**
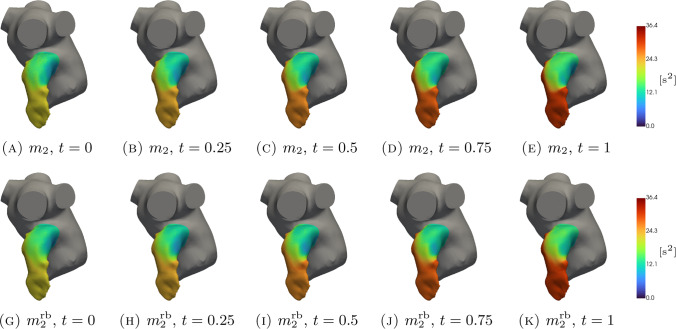
Newtonian case: qualitative comparison between FOM (top) and ROM (bottom) solutions at different times for $m_2$

**Fig. 8:**
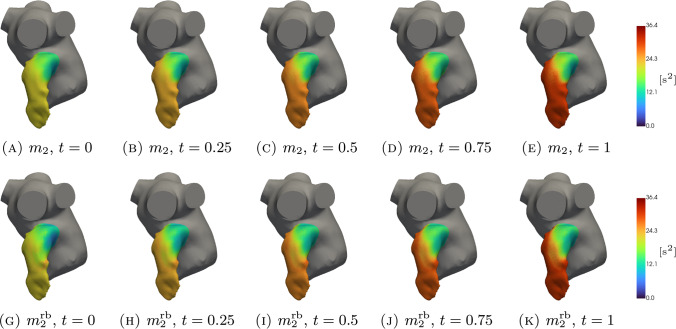
Casson’s case: qualitative comparison between FOM (top) and ROM (bottom) solutions at different times for $m_2$

**Fig. 9:**
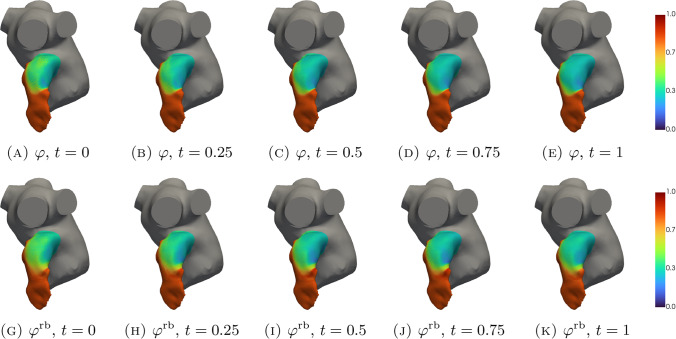
Newtonian case: qualitative comparison between FOM (top) and ROM (bottom) solutions at different times for $\varphi$

**Fig. 10:**
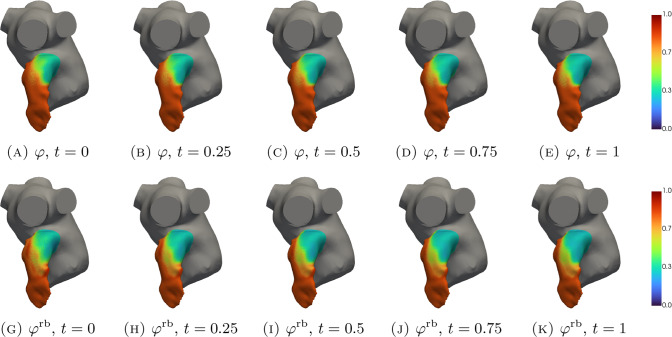
Casson’s case: qualitative comparison between FOM (top) and ROM (bottom) solutions at different times for $\varphi$

**Fig. 11:**
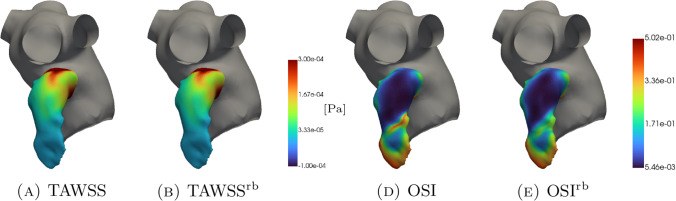
Newtonian case: qualitative comparison between FOM and ROM solutions for $\textrm{TAWSS}$ (panels A and B) and $\textrm{OSI}$ (panels D and E)

**Fig. 12:**
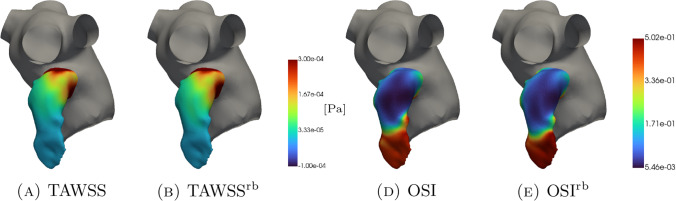
Casson’s case: qualitative comparison between FOM and ROM solutions for $\textrm{TAWSS}$ (panels A and B) and $\textrm{OSI}$ (panels D and E)

In Figs. 5, 6, 7, 8, 9, 10, 11 and 12, we show some qualitative comparisons between the FOM and ROM solutions for the Newtonian and Casson’s cases. The plots refer to the test parameters values $\varvec{\mu }^N_3$ and $\varvec{\mu }^C_{1}$. Figures 5, 7 and 9 (6, 8 and 10) show the results obtained for the variables $m_1$, $m_2$ and $\varphi$, respectively, for the Newtonian (Casson) case. For each variable, the FOM simulations (top plots) are very similar to the ROM ones (bottom plots) as expected by the accuracy analysis based on the relative error carried out above. Furthermore, by looking at the patterns obtained, some speculations of clinical interest can be made. Both models reveal an higher momentum $m_1$ in the terminal region of the LAA. Also the washout $\varphi$ and $m_2$ are characterized by a larger magnitude on the tip of the appendage. Therefore, we can argue that a higher residence time at the end of the LAA is shown. Note that, although in the Newtonian case a slightly greater variation between the base of the appendage and its tip is shown for all the variables under consideration (compare Figs. 5 and 6, Figs. 7 and 8, Figs. 9 and 10), it would seem that for this study case the introduction of Casson’s model is not justified. Finally, in agreement with the trend of the relative errors, also the qualitative comparisons of the Casson’s case reveal a worse reconstruction of the reduced solution at $t=0$.

For what concerns time independent indices, in Fig. 11 and 12, we see that the TAWSS distribution is very well reconstructed by the ROM. As expected, it shows greater values where the blood age is lower and the computations provided by Newton and Casson’s models are very similar. Furthermore, a more pronounced difference between the two rheologies can be found in the OSI distribution.

However, deeper investigations should be conducted, also in direct contact with medical doctors, to establish whether the differences shown by Newton and Casson’s models may be substantial or negligible.

### Computational cost

In Table 1, we report the computational time taken by the FOM and the ROM. In this case, we refer to the entire domain (and not only to the LAA region) in order to provide a fair comparison. All the simulations have been run on the SISSA HPC cluster Ulysses (200 TFLOPS, 2TB RAM, 7000 cores), the FOM ones in parallel using 16 processors while the ROM ones by using one processor only. Each FOM simulation takes roughly 3.75 h in terms of wall time, or 60 h in terms of total CPU time, while the online phase only needs a few seconds. Thus, the speed-up is of the order of $10^5$ and the ROM is able to practically work in a real-time way. In Table 1, we also report the estimation of the time required for the computation of the POD basis and the RBF interpolation for sake of completeness. Such results are very promising and could push toward the transfer of ROM techniques in hospitals and surgery rooms by means of the development of user-friendly digital platforms to be accessed with portable devices, such as a smartphone or a tablet. In this context, we have designed the ATLAS project that allows computations to be run from standard web browsers. Further details could be found in Girfoglio et al. (2021).

**Table 1:** CPU time taken by the offline and the online phases related to the whole computational domain

Model	Offline time			Online	Speed-up
	FOM	POD	RBF	time	
Newtonian	60 h	35 min	11 s	7 s	1e+05
Casson’s	60 h	60 min	3 s	9 s	1e+05

## Conclusions and perspectives

A data-driven ROM based on POD-RBF technique is adopted in this work for the analysis of the blood flow in a patient-specific domain of LA when AF occurs. Such approach extracts a reduced basis space from a proper set of high-fidelity solutions via POD and adopts RBF to compute the map between parameter space and reduced coefficients. The Newtonian and the Casson’s models for the rheology of the blood are employed and compared. We consider a physical parametric framework involving the scaling factor of the cardiac output (both for Newtonian and Casson’s case), the plasma viscosity and the hematocrit (for the Casson’s case).

After an expensive offline phase, the POD-RBF approach demonstrated to be able to provide clinically relevant blood flow predictions for the problem at hand at a considerably lower computational cost. This indicates that in perspective such computational tool could be used in hospitals and surgery rooms to support the medical doctors. From a clinical point of view, the results showed that the distribution of the mean blood age is higher on the tip of LAA (regardless of whether the Newtonian model or the Casson’s model is employed) because this is the most isolated zone of the computational domain and the harder to wash.

The ROM described in the paper can be applied to various geometries of left atrium, with similar results, because POD-RBF is a general tool. However, the extrapolation to unknown geometries requires a geometrical parameterization, which is not included in this work, as it typically involves greater complexity and data requirements compared to physical parameterization. In perspective, an improvement of considerable relevance could be the exploration of the performance of the POD-RBF approach in a geometrical parametric setting by extending to our case study what carried out in Siena et al. (2023). Also, the influence of variable inflow conditions including flow splitting in the pulmonary veins will be studied. Another possible follow-up is represented by the development of a hybrid ROM Girfoglio et al. (2023); Hijazi et al. (2020), where a projection-based approach is introduced for the primal quantities of the problem, i.e., velocity and pressure. This introduces some physical constraints in the ROM model making it more accurate. Finally, nonlinear ROMs (see, e.g., Fresca et al. (2021); Milano and Koumoutsakos (2002); Lee and Carlberg (2020)) could be investigated.

## Figures and Tables

**Fig. 13:**
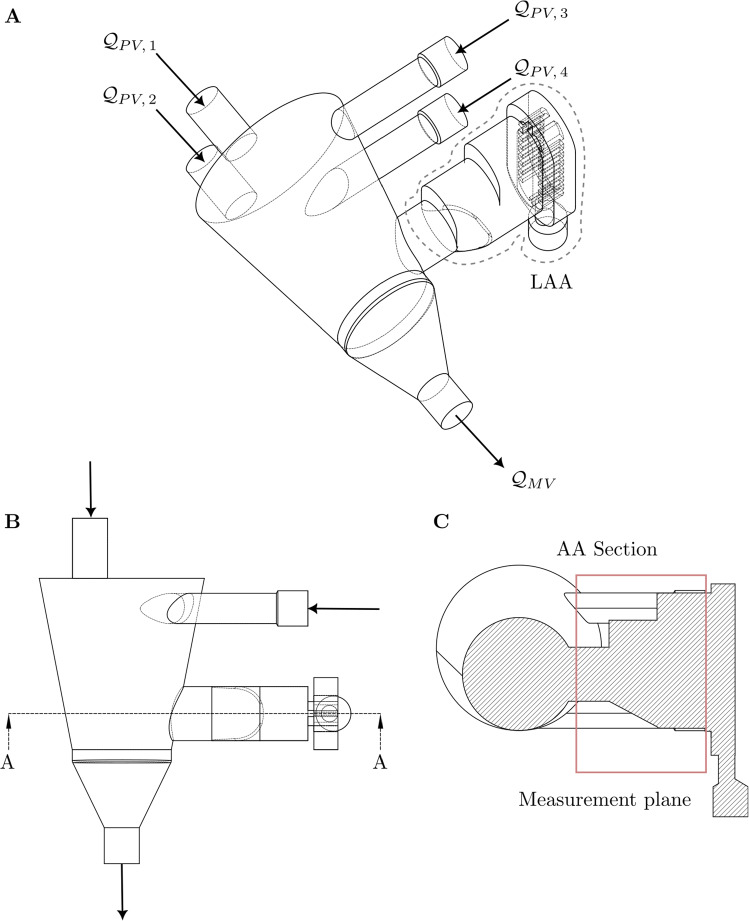
Description of validation experiment taken from Dueñas-Pamplona et al. (2021). **A**: Isometric view of the in vitro LA with inflows $\mathcal {Q}_{PV,\,i}$, mitral valve outflow $\mathcal {Q}_{MV}$ and LAA volume highlighted; **B**: Front view of the in vitro LA; **C**: Detailed view of the measurement section and plane at the in vitro LAA

**Fig. 14:**
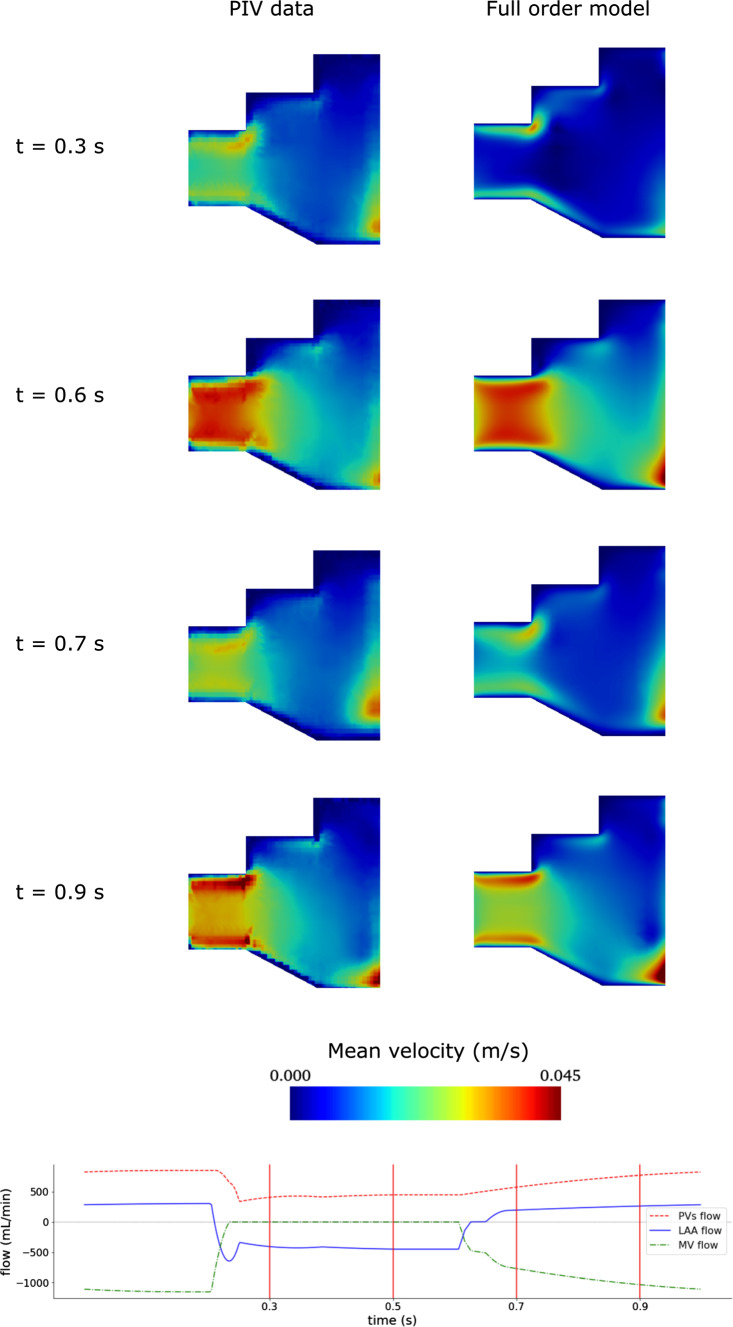
Comparison of mean velocity field obtained by experimental measurements (PIV, Dueñas-Pamplona et al. (2021)) Vs. OpenFOAM simulation data (top) and boundary conditions (bottom)

## Supporting materials

### Model validation

Model validation is a critical step in the development of computational models and simulations, particularly in engineering and scientific research. Model validation involves comparing the model’s predictions to experimental or observational data, verifying that the model accurately captures the behavior of the system under study. In this work, the FOM is validated against the experimental benchmark reported by Dueñas-Pamplona et al. (2021). In that work, an in vitro model mimicking a real left atrium geometry working with a blood mimicking fluid is measured by particle image velocimetry (PIV). Figure 13 shows the experimental geometry and the measurement plane employed. Figure 14 shows the experimental measurements of the mean velocity in the in vitro model (top of the figure) along with boundary conditions (bottom of the figure) for mass flow rates and the predictions of our FOM implementation. Mean velocity is well predicted by the FOM, which is in line with the results provided in the benchmark paper Dueñas-Pamplona et al. (2021), with an absolute average deviation of around 20% from the experimental data. In fact, current OpenFOAM implementation gives almost identical results to those obtained by the authors in that reference.

### Mesh convergence analysis

Mesh convergence analysis is a crucial step in verifying a CFD code, for ensuring that a CFD simulation is properly resolved and that the numerical results are reliable. The Grid Convergence Index (GCI) Roache (1998) is used here since it is a widely accepted method for assessing the mesh convergence of a CFD simulation. The GCI provides an estimate of the order of accuracy and the uncertainty associated with the numerical solution obtained from a given mesh. The GCI is calculated by comparing the solutions obtained from two or more grids with different mesh sizes.

Table 2 shows the result of a GCI study on four different grids for the patient-specific geometry at hand (see Fig. 1), taking a point located at the geometric center of the ostium as sample point. Mean velocity is evaluated in such grid point for four different grid spacing, assuming in all cases a time step of $10^{-3}$ s and running the simulation for 5 complete cycles. Results show the solution on Grid 2 is mesh independent; therefore, this grid size is used for all the simulations in this study.

**Table 2:** Grid convergence study over 4 grids.

	$\bar{v}$	$N_{h}$	*r*	$\text {GCI}$	$\text {GCI}_{\text {asymptotic}}$	*p*	$\bar{\textbf{v}}_{\text {extrapolated}}$
Grid 1	4.755e–02	1230787	1.3	7.38%	1.014	1.31	5.04e–02
Grid 2	4.63e–02	542560	1.5	10.71%			
Grid 3	4.38e–02	174370	–	–			
Grid 2	4.634e–02	542560	1.5	20.83%	1.678	0.75	5.41e–02
Grid 3	4.38e–02	174370	1.3	46.49%			
Grid 4	4.68e–02	88564	–	–			

$\bar{\textbf{v}}$ represents the mean velocity at a point located in the ostium of the LAA and $\bar{\textbf{v}}_{\text {extrapolated}}$ its extrapolated value. $N_{h}$ is the number of grid elements, *r* the refinement ration between two successive grids. $\text {GCI}$ is the grid convergence index in percent and its asymptotic value is provided by $\text {GCI}_{\text {asymptotic}}$, where a value close to unity indicates a grid independent solution. The order achieved in the simulation is given by *p* Roache (1998)
